# Transapical Implantation of a 2nd-Generation JenaValve Device in Patient with Extremely High Surgical Risk

**DOI:** 10.1155/2015/458151

**Published:** 2015-08-04

**Authors:** Juan Mieres, Marcelo Menéndez, Carlos Fernández-Pereira, Miguel Rubio, Alfredo E. Rodríguez

**Affiliations:** Cardiac Unit and Cardiovascular Surgery Department, Otamendi Hospital, Azcuènaga 870, C1115AAB Buenos Aires, Argentina

## Abstract

Transcatheter Aortic Valve Replacement (TAVR) is performed in patients who are poor surgical candidates. Many patients have inadequate femoral access, and alternative access sites have been used such as the transapical approach discussed in this paper. We present an elderly and fragile patient not suitable for surgery for unacceptable high risk, including poor ventricular function, previous myocardial infarction with percutaneous coronary intervention, pericardial effusion, and previous cardiac surgery with replacement of mechanical mitral valve. Transapical aortic valve replacement with a second-generation self-expanding JenaValve is performed. The JenaValve is a second-generation transapical TAVR valve consisting of a porcine root valve mounted on a low-profile nitinol stent. The valve is fully retrievable and repositionable. We discuss transapical access, implantation technique, and feasibility of valve implantation in this extremely high surgical risk patient.

## 1. Introduction

Transcatheter Aortic Valve Replacement (TAVR) was introduced as a therapeutic option in treatment of severe aortic stenosis (AS) in elderly patients who are poor candidates for conventional surgical aortic valve replacement.

Since TAVR was first introduced [1], thousands of patients have been treated worldwide and their feasibility and safety have been demonstrated in randomized trials and large observational studies [2, 3]. Different prosthesis designs have been used, particularly two of them in the majority of the cases [4].

Femoral approach has been the most frequent access site, although vascular access site complications and cerebrovascular accident have been reported with this approach just as a significant increase in 30-day mortality [5].

For this reason other access sites are being used, such as transapical, carotid, subclavian, and transaortic, mainly when femoral approach is not feasible [6].

The purpose of this presentation is to report results achieved in a high risk patient with AS using transapical approach and implantation of second-generation JenaValve device [7, 8].

## 2. Case Presentation

83-year-old female presented hypertension, high cholesterol, and previous mechanical mitral valve replacement for severe mitral insufficiency in 1992.

Patient also has severe coronary artery disease treated with three bare metal stents (BMS) to left anterior descending artery (LAD) three years ago and non-ST elevation anterior myocardial infarction three months ago.

She was, at the time, symptomatic with multiple hospitalizations for heart failure and severe AS. In the last month, she had presented progressive dyspnea from functional classes II to IV NYHA associated with paroxysmal nocturnal dyspnea and orthopnea and lower extremities edema worsening in the 48 hours prior to hospital admission.

**Figure 1:**
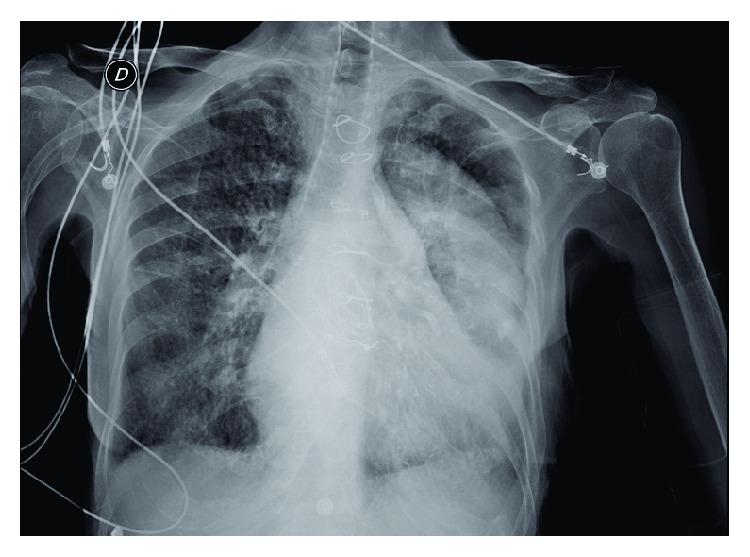
Chest X-ray showing pleural effusion.

At physical examination she was lucid, normotensive, afebrile, and tachypneic with bilateral crackles to vertex and jugular engorgement 3/3 with hepatic jugular reflux; chest X-ray showed pleural effusion in left lung and signs of congestive heart failure (Figure 1).

EKG showed left anterior hemiblock, anterior myocardial infarction sequel, and negative T-waves in precordial and lateral leads.

**Figure 2:**
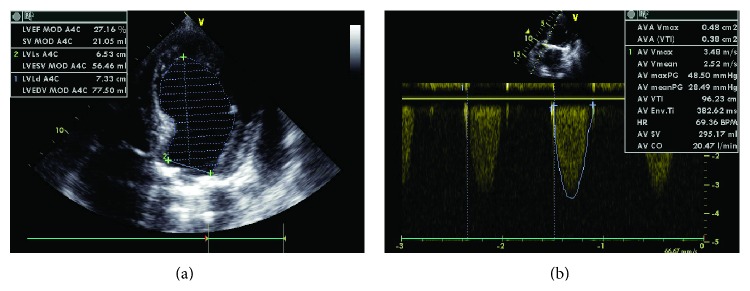
(a) Severe impairment of the left ventricular systolic function with pericardial effusion. (b) Severe aortic stenosis.

Transthoracic echocardiogram presented akinesia of the apical segments and mid anterior septum and severe impairment in left ventricular systolic function and ejection fraction (EF) of 27% with mild to moderate pericardial effusion (Figure 2(a)).

There was a mechanical prosthesis in mitral position without signs of malfunction and we confirmed the presence of severe AS with aortic valve area of 0.5 cm^2^ (Figure 2(b)).

Coronary angiography was performed, showing severe intrastent restenosis in mid portion of LAD and mild lesions on the other arteries. Taking into account the high surgical risk defined by our Heart Team, Euroscore II 23.1, and STS with a predicted morbidity or mortality of 50.2%, a conventional aortic valve replacement was ruled out and TAVR planned with transapical approach selected.

Reasons for this selection were the small size of both iliac arteries, <6 mm, and the presence of previous cardiac surgery with mitral valve prosthesis with a short distance between mitral and aortic rings.

First, as part of the strategy, a PCI to LAD with cutting balloon Boston Scientific (Marlborough, MA, USA) plus plain balloon angioplasty Ryujin Terumo (Somerset, NJ, USA) and balloon Quantum Maverick Boston Scientific (Marlborough, MA, USA) was performed without complications 72 hours previously to the percutaneous valve implantation.

**Figure 3:**
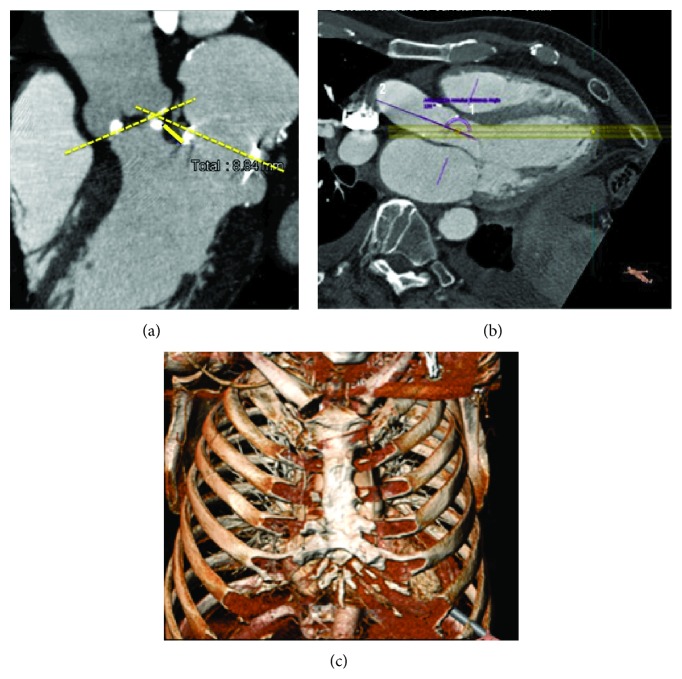
(a) 3D computer tomography images showing distance between aortic and mitral ring, (b) anterograde annulus entrance angles, and (c) place of transapical and 3D reconstruction of intercostal space site access.

Access site to the apical wall of left ventricle was previously guided by a 3D computer tomography angiogram, anterograde annulus entrance angle of 158°, and distance annulus to aortic arc of 68 mm (Figures 3(a), 3(b), and 3(c)). The patient had an annulus of 21.7 mm and a perimeter of 69.1 mm, so we selected a JenaValve size of 23.

**Figure 4:**
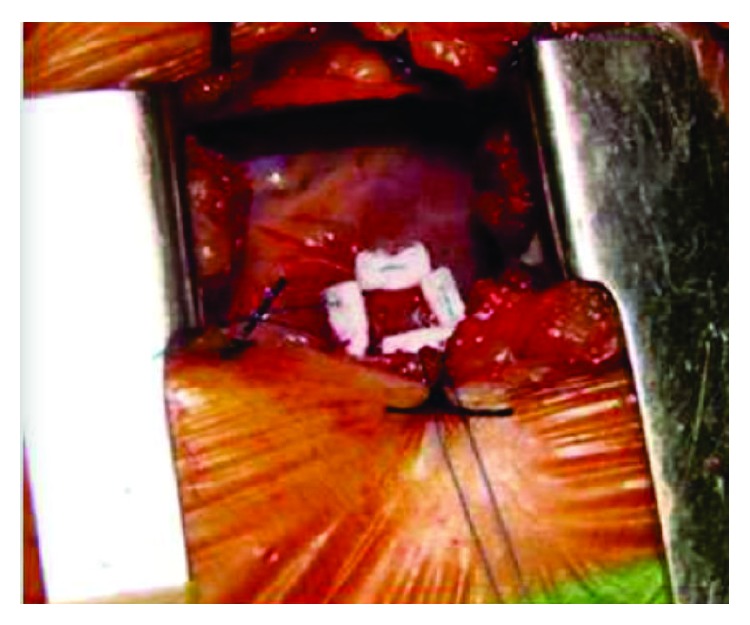
Transapical access with a mini thoracotomy.

**Figure 5:**
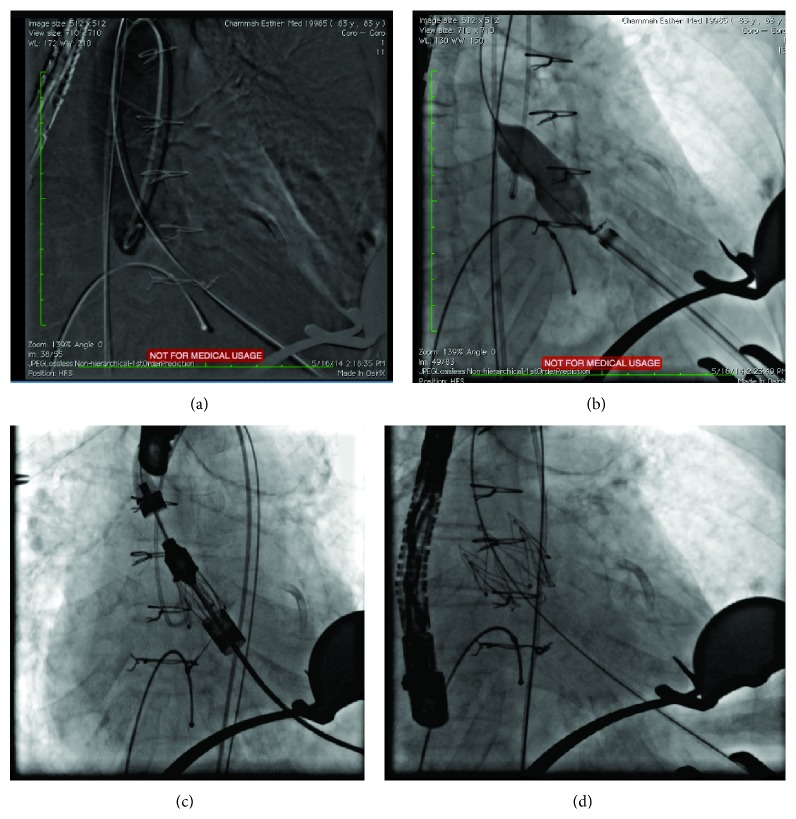
(a) Extra shift Amplatz 0,35 guide wire was deployed in descendent aorta to give extra backup support. (b) Aortic valvuloplasty with 22 mm balloon. ((c), (d)) A 32-French size delivery with its JenaValve device can be done, and a valve number 23 is implanted.

A transapical access with a mini thoracotomy from fifth intercostal space was performed (Figure 4). We proceeded with transapical approach with initial insertion of a puncture needle using a Terumo (Somerset, NJ, USA) 0.35 J shape guiding wire to cross aortic valve through aortic arc; usually the aortic valve using this anterograde approach is crossed very easily independently of the degree of valvular stenosis. Afterwards a 6F sheath was implanted and a right coronary catheter Terumo (Somerset, NJ, USA) was crossing through the aortic valve, in direction of aortic arc and descendent aorta. At this point we change the wire guide and an extra shift Amplatz (Boston Scientific, Marlborough, MA, USA) 0,35 guide wire was deployed in descendent aorta to give extra backup support (Figure 5(a)).

Through the Amplatz guide an 18 Fr sheath crossing the left ventricular apex was deployed and aortic valvuloplasty with 22 mm Zelos PTA, Balloon Catheter (Ettlingen, Germany), was performed under temporary pacemaker (Figure 5(b)). Immediately after valvuloplasty, 18 Fr sheath was removed and in spite of poor left ventricular function and previous cardiac surgery, a 32-French size was successfully delivered and the JenaValve (Guerickestraße, München, Germany) device was implanted and a valve number 23 placed using a three-step deployment system (Figures 5(c) and 5(d)) under 3D fluoroscopy and transesophageal echo. After JenaValve implantation, the device was retrieved easily under simultaneous 150 pacing beats per minute.

**Figure 6:**
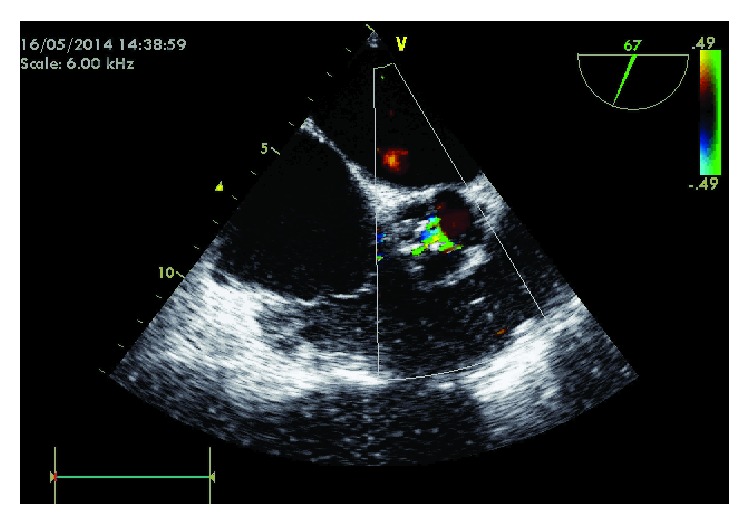
No residual valve leak, with mild valve gradient and an aortic valve area of 1.42 cm^2^ after implantation.

Patient had no residual valve leak, with mild valve gradient and an aortic valve area of 1.42 cm^2^ after implantation (Figure 6).

During the first 24 hours, patient presented low cardiac output and oliguria requiring dobutamine and intravenous furosemide.

With EKG showing no changes, temporary pacemaker was withdrawn 24 hours after valve implantation. Patient was discharged at day 6, asymptomatic, with 40 mg of furosemide, 75 mg clopidogrel, 100 mg aspirin, and 3.25 mg of bisoprolol daily.

**Figure 7:**
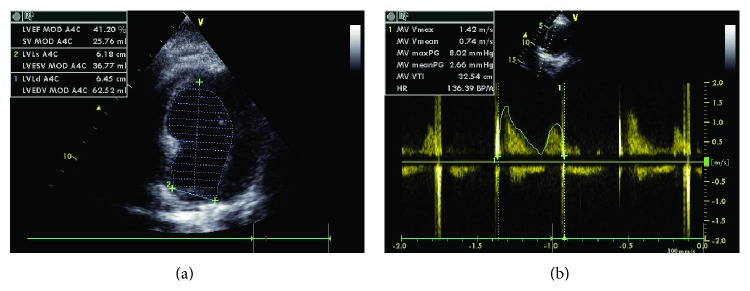
(a) Hospital discharge transthoracic ECHO showed a significant improvement of left ventricular EF, 41%, (b) 1.42 of aortic valve area and Vmax of 2.66.

At time of hospital discharge, transthoracic echocardiogram showed a significant improvement of left ventricular EF, 41% with an aortic valve area of 1.42 cm^2^ (Figure 7).

Four months after implantation patient presented progressive chest pain with ischemic T-waves in lateral leads and was treated medically.

At one year follow-up, patient is alive, in functional classes I-II with an aortic valve area of 1.4 cm^2^.

## 3. Discussion

We are presenting a case of severe AS with a critical valve area of 0.5 cm^2^ in a patient with high risk morbidity or mortality defined by Heart Team as “elderly female with previous cardiac surgery with mitral valve replacement, severe coronary artery disease, previous myocardial infarction, poor left ventricular EF, pleural and pericardial effusion and severe congestive heart failure.” The case was successfully treated using a percutaneous transapical implantation of a second-generation JenaValve [9].

TAVR significantly improves survival and functional class in elderly patients with high surgical risk. Femoral approach is the most frequent access for valve implantation; however there are several circumstances where other access sites are chosen [10].

In this case, presence of small iliac arteries, previous mitral surgical valve implantation, and small distance between mitral and aortic rings almost contraindicated access needed for the other aortic valve device available in Argentina.

The use of CT angiogram previous to the procedure facilitates a better selection of the access site for transapical approach and selects the right coaxial approach of the left ventricle and aortic arch.

Pacing left ventricle during device retrieval allows a softer repossession of the 32 Fr JenaValve sheath without damage to the left ventricle wall in spite of previous valve replacement with pericardial effusion present in our patient.

Even though several large registries have shown that femoral access had lower incidence of morbidity or mortality risk compared to transapical access, the same registries also showed that patients with transapical approach also had significant poor baseline comorbidities associated with high procedural risk [11]. Furthermore, the same registries also showed that apical access was associated with lower incidence of vascular complications and perivalvular aortic leak, both complications linked with late follow-up mortality [12].

A multicenter registry in Europe with the JenaValve device reported high procedural success and very low incidence of cerebrovascular complications (1.1%) with residual moderate aortic regurgitation in only 0.6% of more than 150 patients. In this series, 6-month freedom from cardiovascular death was 90.6% [13]. The second-generation self-expanding JenaValve device is available to be used percutaneously in patients with AS and also with aortic insufficiency [14].

In our initial experience with this device [15] we reported 30-day results from the first 18 patients with a survival of 100% of them without vascular, cerebrovascular, or coronary complications. No patients had residual aortic insufficiency and none required permanent pacemaker. All procedures were performed together by all members of the Heart Team: a cardiac surgeon opened and closed the left ventricle and an interventional cardiologist performed the entire process of percutaneous valve implantation.

Finally, cutting and plain balloon angioplasty to mid portion LAD stenosis was selected as PCI strategy taking into account the short period of time between PCI and aortic valve implantation and the fact that the lesion was located at mid portion of LAD artery presenting an area with previous large anterior myocardial infarction.

In conclusion, we are reporting a case with clinical indication of percutaneous aortic valve replacement in a patient at high risk for femoral access, successfully treated with implantation of a second-generation JenaValve device. To our knowledge, only a few JenaValve patients have been reported in medical literature but none with the amount of comorbidities as our patient [16–19].
